# Facilitation and interference are asymmetric in holistic face processing

**DOI:** 10.3758/s13423-024-02481-9

**Published:** 2024-03-04

**Authors:** Haiyang Jin, Luyan Ji, Olivia S. Cheung, William G. Hayward

**Affiliations:** 1 Department of Psychology, Zhejiang Sci-Tech University, Hangzhou, China; 2 Department of Psychology, New York University Abu Dhabi, Abu Dhabi, United Arab Emirates; 3 Department of Psychology, Guangzhou University, Guangzhou, China; 4 Center for Brain and Health, NYUAD Research Institute, New York University Abu Dhabi, Abu Dhabi, United Arab Emirates; 5 Department of Psychology, Lingnan University, Hong Kong, China

**Keywords:** Face perception, Holistic processing, Composite face task, Facilitation, Interference

## Abstract

A hallmark of face specificity is holistic processing. It is typically measured by paradigms such as the part–whole and composite tasks. However, these tasks show little evidence for common variance, so a comprehensive account of holistic processing remains elusive. One aspect that varies between tasks is whether they measure *facilitation* or *interference* from holistic processing. In this study, we examined facilitation and interference in a single paradigm to determine the way in which they manifest during a face perception task. Using congruent and incongruent trials in the complete composite face task, we found that these two aspects are asymmetrically influenced by the location and cueing probabilities of the target facial half, suggesting that they may operate somewhat independently. We argue that distinguishing facilitation and interference has the potential to disentangle mixed findings from different popular paradigms measuring holistic processing in one unified framework.

## Introduction

The ability to make judgments about the human face is integral to human social interaction. Through the study of face perception and its underlying neural correlates, multiple face-related processing indicators have been uncovered, which have, in turn, deepened our understanding of face processing (e.g., Poltoratski et al., 2021). One of the most important theoretical constructs for face perception is *holistic processing*—that is, the integrated processing of multiple facial parts (Farah et al., 1998; Hayward et al., 2013; Maurer et al., 2002; Rossion, 2013). Evidence for this claim comes from numerous studies demonstrating that processing of one facial part is often influenced by the other facial parts (Amishav & Kimchi, 2010; Hayward et al., 2016; Meinhardt-Injac et al., 2014; Richler et al., 2015; Tanaka & Sengco, 1997). However, theoretical clarity for the construct of “holistic processing” is lacking, as noted by Rossion (2008), who proposes a definition of holistic processing as “the simultaneous integration of the multiple features of a face into a single perceptual representation,” yet notes that most empirical evidence “essentially shows that faces features are *interdependent*” (p. 275). Perhaps not surprisingly, given this lack of theoretical clarity, evidence from studies purporting to investigate holistic processing have shown a variety of results. In this paper, we seek to propose a new framework for understanding the nature of interdependent processing of facial features, with the goal to gain a better understanding of what is meant by the construct of “holistic processing” in order to allow for the development of a more comprehensive and testable theory.

**Fig. 1:**
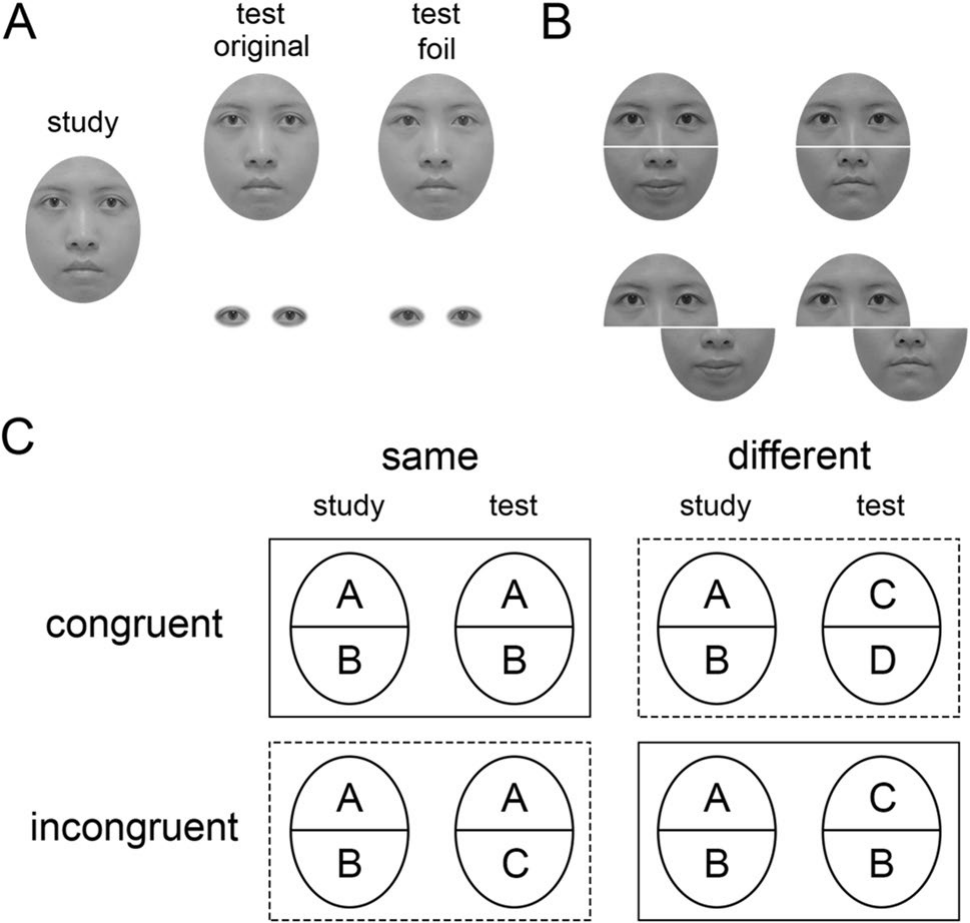
Experimental designs of different tasks used to assess holistic face processing. **A** The part–whole (PW) task. After studying a face, test stimuli are either two versions of the study face varying in one feature only (top row) or two versions of a single feature (bottom row). **B** Example stimuli used in the standard composite face task (SCF) and complete composite face task (CCF). Both rows display two face composites, where the top facial halves are the same and the bottom halves are different. Composites may be aligned (top row) or misaligned (bottom row). **C** Schematic composite pairs in the SCF and CCF, showing Congruency when top halves are the target stimulus, and letters denote identity of the original faces. Left and right columns are trials where the target (i.e., top) halves are the same or different. The first and second rows are trials where the relationships between target and irrelevant (i.e., bottom) halves in study and test faces are congruent and incongruent. (1) The CCF consists of all the four trial types, where the composite effect is typically characterized by the interaction between Congruency (congruent vs. incongruent) and Alignment (aligned vs. misaligned). (2) Different from the CCF, the SCF only includes the same-incongruent and different-congruent trials, as shown in the two dashed line rectangles. The composite effect, by contrast, is indexed by the differences in “same(-incongruent)” trials between the aligned and misaligned conditions. (3) The trials in the PW task can be characterized as being equivalent to the same-congruent and different-incongruent trial types, as outlined with the solid rectangles (for more information, see the General Discussion)

Holistic face processing is most commonly measured by two popular paradigms: the composite task and the part–whole task. In the composite task^1^ (Hole, 1994; Richler & Gauthier, 2014; Rossion, 2013; Young et al., 1987), participants are shown two composite faces (Fig. 1B), which are created by aligning the top and bottom facial halves from different individuals, and are instructed to determine if particular halves of the two faces are identical while ignoring the other halves. Results suggest, for example, that the same top half looks different when it is aligned with different bottom halves; however, this effect is reduced when the two halves are misaligned. The task stands in contrast to the part–whole task (Tanaka & Farah, 1993; Fig. 1A): participants first learn a study face and then are given two isolated features (e.g., eyes), one of which is in the original face, or two whole faces which are identical except for one feature. Participants are instructed to choose which of the two isolated features or two whole faces is the learned stimulus. Identification performance is typically better for whole faces than for parts, even though the additional information in the whole faces is identical (Tanaka & Farah, 1993; Tanaka & Simonyi, 2016). These paradigms provide important evidence that processing of one face part is influenced by the other parts, supporting the holistic face processing hypothesis.

As noted above, these paradigms are widely referred to in the literature as measuring a construct that is called “holistic face processing,” but this term is so vague that the extent to which these tasks measure the same construct is difficult to determine. Several studies have failed to find significant correlations between the part–whole and the standard composite tasks (e.g., Boutet et al., 2021; Rezlescu et al., 2017; Wang et al., 2012); further, it appears that distinct neural mechanisms may underlie these tasks (Li et al., 2017, 2019). DeGutis and colleagues (DeGutis et al., 2013) observed significant correlations between the part–whole task and the complete composite task when regressions, but not subtractions, were employed to calculate the holistic effects.^2^ This finding suggests that the correlation between the part–whole and complete composite effect is not reliably observed. Few studies have explored the relationships between the standard and complete composite tasks, probably because researchers usually only choose one of them within a single study. One key exception is Richler and Gauthier (2014), who performed a meta-analysis to explore their potential relationships and did not observe any significant correlations between the effect sizes in standard and complete composite tasks, even though the former were a subset of the latter. Overall, the relations among the effects measured by these paradigms are mixed but the evidence seems to suggest that they do not measure the same aspect of holistic face processing.

To make progress in this field, we examined the aspects of holistic processing that are measured by these different paradigms. Here, we re-visit them in terms of the nature of the interdependence between the target and irrelevant facial parts in each task. In the part–whole task, the “redundant” facial parts *facilitate* the judgment of the target feature (Tanaka & Simonyi, 2016). In the standard composite task, the bottom halves are always different between study and test faces and *interfere* with recognition of the aligned same top halves (Hole, 1994; Young et al., 1987). In the complete composite task, both the top and bottom facial halves may be identical or different on each trial. When participants make identity judgments about the target halves of two composites, the irrelevant halves *facilitate* or *interfere* with target performance based on whether the identity relationship between the irrelevant halves is identical to (congruent) or different from (incongruent) that of the target halves. In summary, different tasks that purport to measure holistic face processing can be differentiated by whether irrelevant parts lead to *facilitation* (as in the part–whole task and congruent trials of the complete composite task) or *interference* (as in the standard composite task and incongruent trials of the complete composite task).

It is not immediately clear whether these two behavioral phenomena—facilitation and interference—originate from the same aspect of holistic processing. If they result from the same aspect, we should observe similar results on both facilitation and interference from the same manipulations (i.e., the effects vary symmetrically; conditions leading to greater facilitation in one task also lead to greater interference). Alternatively, if facilitation and interference stem from different aspects of holistic processing, an asymmetry between the two effects should be observed. Asymmetries in these effects would help explain why different tasks showed poor correlations in previous studies. To investigate this issue, we inspected facilitation and interference in a single holistic face processing paradigm to exclude the influence of potential confounds when using multiple paradigms, such as task formats (e.g., two-alternative-force-choice in part–whole task vs. sequential matching in composite tasks) and response bias (Richler et al., 2011; Rossion, 2013; Rossion & Retter, 2015). Specifically, we investigated facilitation and interference observed in congruent and incongruent trials in the complete composite task. Experiment 1 examined both facilitation and interference effects, and their dependency on the location of the target halves (top vs. bottom). In Experiment 2, the probability of cueing particular target halves was manipulated (e.g., the top half was cued 75% of trials in one session compared to 25% in a second session) to examine the impact of strategic processes on facilitation and interference.

With the use of the complete composite task, we followed its main proponents, Richler and Gauthier (2014), and adopt their definition of holistic processing reflecting “obligatory encoding of all object parts because a strategy of attending to all parts cannot be ‘turned off’.” More specifically in the complete composite task, holistic processing is measured by “the failure of selective attention” (i.e., the extent to which participants could not ignore the influence of the irrelevant parts; Richler & Gauthier, 2014; see also Farah et al., 1998). If faces are processed holistically, we should observe greater influence of irrelevant parts on target parts for aligned faces—that is, the larger congruency effect (better performance for congruent compared to incongruent conditions) for aligned relative to misaligned faces (i.e., the interaction between Congruency and Alignment; see more below). Moreover, regarding our specific observation of interest, if there is facilitation, we should observe better performance for aligned congruent faces compared to misaligned congruent faces. If there is interference, we should observe worse performance for aligned incongruent faces relative to misaligned incongruent faces. Any perceptual or cognitive effect that stems from nonholistic processing or is not specific to aligned faces should not affect these observations of facilitation or interference since such effects would be expected to influence aligned and misaligned stimuli identically.

## Experiment 1

### Methods

#### Participants

We employed G*Power (Faul et al., 2009) to plan the sample size. It suggests that at least 31 participants would suffice for the statistical power of 95% with the alpha of 0.05 and the partial eta square of 0.32, which was the average effect size for the composite effect estimated by Richler and Gauthier (2014).

Thirty-two Chinese students (15 females and 17 males, mean age = 24.18 years) from the University of Hong Kong participated and were compensated with one course credit or 60 HKD. Participants gave written informed consent prior to the experiment and reported that they were right-handed and had normal or corrected-to-normal vision. The study protocol was conducted in accordance with the Declaration of Helsinki and approved by the local ethics committee.

#### Stimuli

**Fig. 2:**
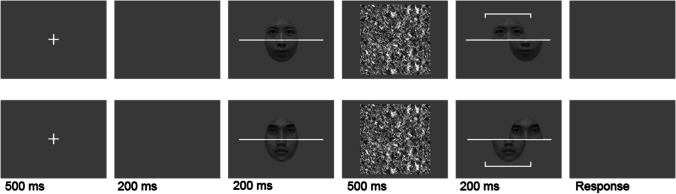
Trial sequence of the composite task in Experiments 1 and 2. Example female and male composites are shown in the top and bottom panels, respectively. On each trial, participants judged if the target halves (cued with white bracket on the test faces) were identical between the study and test faces

Photos of 40 Chinese (20 females and 20 males) faces with neutral expression were converted to greyscale and trimmed into an oval shape with external features (e.g., hair and ears) excluded. The luminance and contrast were controlled using the SHINE toolbox (Willenbockel et al., 2010). Each face was divided into one top and one bottom half from the middle of the face (Fig. 2). A three-pixel white line separated the two face halves; thus, the top and bottom facial halves were unambiguous to participants. To render composite faces more biologically plausible and retain the randomization of creating composites from facial parts, we divided the stimuli into ten sets, with each containing four different individuals of the same gender. The top and bottom facial halves in the same set were randomly combined to form composite faces. The study and test composites on each trial were from the same stimulus set. We used the complete design with all trial types of composites (Fig. 1C), including misaligned conditions. The study and test faces were presented at the center of the screen with a homogenous gray background. The top and bottom halves of the study faces were always aligned. The test faces in half of the trials were aligned and in the other half of the trials were misaligned, where the irrelevant facial half shifted half of the face width to the right. Aligned and misaligned composite faces subtended 5.59° × 7.24° and 8.39° × 7.24° of visual angle, respectively.

#### Apparatus and procedure

Participants sat approximately 50 cm in front of a 17-inch flat screen with a refresh rate of 60 Hz. The task was administrated with Psychtoolbox (Kleiner et al., 2007). On each trial (Fig. 2), a fixation cross appeared at the center of the screen for 500 ms, followed by a blank for 200 ms, a study face for 200 ms, a mask for 500 ms, then a test face and a white bracket were presented simultaneously for 200 ms. The white bracket was presented randomly either above or below the face, indicating either the top or bottom half of the face was the target. Participants were instructed to judge whether the cued target parts were identical between the study and test faces by pressing one of two keys, with the response mapping counterbalanced across participants. Both accuracy and speed were emphasized. The next trial started 1,000–1,200 ms after a key press. The experiment lasted around 50 min.

This experiment involved four within-subject factors: Target (top vs. bottom), Congruency (congruent vs. incongruent), Alignment (aligned vs. misaligned), and Correct Response (same vs. different). There was a total of 640 trials, with 40 trials in each condition. Before the actual experiment, participants performed 32 trials with line-drawing stimuli with the same proportion of trials in each condition.

#### Open science statements

The analysis codes and datasets generated and/or analyzed for this study are available online (https://osf.io/yhm3s/). The experiments were not preregistered.

#### Data analysis

All analyses were conducted in R (Version 4.0.5) and RStudio (Version 1.4) on a local computer, except for model fitting, which was carried out in R (Version 3.6) on the High-Performance Computing platform at New York University Abu Dhabi. We tidied up the data with the “tidyverse” package (Wickham et al., 2019). All trials were included in the analyses except when no response was recorded (one trial each from three participants). To avoid the limitations of analyses of variance (ANOVAs; Aarts et al., 2014; Boisgontier & Cheval, 2016; Kristensen & Hansen, 2004; Quené & van den Bergh, 2004), such as the additional sphericity assumption, we employed generalized linear mixed-effects models (GLMM) to analyze behavior choices and correct response times (RT)^3^ with “lme4” package (Bates, Mächler et al., 2015b) where successive difference contrast coding was applied. Follow-up comparisons were performed using the “emmeans” package (Lenth, 2023) with “asymptotic” methods estimating statistical results.

Although GLMM with the maximal random-effects structure (i.e., the maximal model) is preferred for confirmatory hypothesis testing (Barr et al., 2013), such models usually cannot converge. Thus, we built the random effects in GLMM by following Bates, Kliegl, and colleagues (Bates, Kliegl et al., 2015a) and Matuschek and colleagues (Matuschek et al., 2017). We first removed correlations between random effects in the maximal model, making the zero-correlation-parameter model. Principal component analysis implemented with “rePCA()” function was then used to identify random effects that explained less than 0.1% of the total variances; they were removed from the zero-correlation-parameter model to make the reduced model. Next, the extended model was built by adding back the correlations between random effects in the reduced model. If the extended model did not converge, the random effects that explained less than 1% of total variances were identified by “rePCA()” and removed to make the updated extended model; this step was iterated until an extended model converged. The converged extended model was then compared to the reduced model via “anova()” function and the model that explained the data better (with smaller Akaike information criterion) was used as the optimal model. All follow-up analyses were performed on the optimal model.

For behavioural choices of same–different judgment, signal detection models were implemented by GLMM with binomial error distribution and “probit” link. Fixed effects included Target, Congruency, Alignment, Correct Response, and all their interactions. Random effects in the maximal model included all the by-subject random intercepts and random slopes. The dependent variable was whether participants responded “same” on each trial. The optimal model was then obtained with the above steps. Two sets of analyses were conducted based on the optimal model of behavioral choices, in which sensitivity *d′* was defined as z(hits) − z(false alarms) and “same” in Correct Response was treated as “signal.” First, the composite effects when participants judged the top and bottom halves were examined. The composite effect was tested by (1) the Congruency effect (congruent − incongruent) for aligned faces in sensitivity *d′*—that is, the interaction between Congruency and Correct Response in the aligned condition and (2) the differences of the Congruency effects between the aligned and misaligned conditions in sensitivity *d′*—that is, the interaction between Congruency, Alignment, and Correct Response. Critically, the composite effect would be claimed only when both effects were significant (Jin, 2021). Second, facilitation and interference effects were examined by pairwise comparisons between aligned and misaligned composites in sensitivity *d′*—that is, interaction between Alignment and Correct Response for congruent and incongruent trials separately. These tests were corrected by Sidak methods for four estimates. Specifically, facilitation would be revealed by better performance for congruent trials with aligned than misaligned composites, whereas interference would be revealed by poorer performance for incongruent trials with aligned than misaligned composites.

For RT, we only included trials in which participants responded correctly. Specifically, GLMM with lognormal transformation was applied. Fixed effects included Target, Congruency, Alignment, and all their interactions. Random effects in the maximal model incorporated all the by-subject random intercepts and random slopes. The optimal model was obtained with the same above steps, on which subsequent analyses were performed. The analyses of composite effects, facilitation and interference in RT were similar to those in sensitivity *d′*. In particular, the composite effects of RT would be claimed only when both (1) the Congruency effect for aligned composites and (2) the interaction between Congruency and Alignment were significant. Facilitation and interference effects were tested by pairwise comparisons between aligned and misaligned composites in congruent and incongruent conditions separately where the correction of Sidak methods for four estimates applied.

### Results

**Fig. 3:**
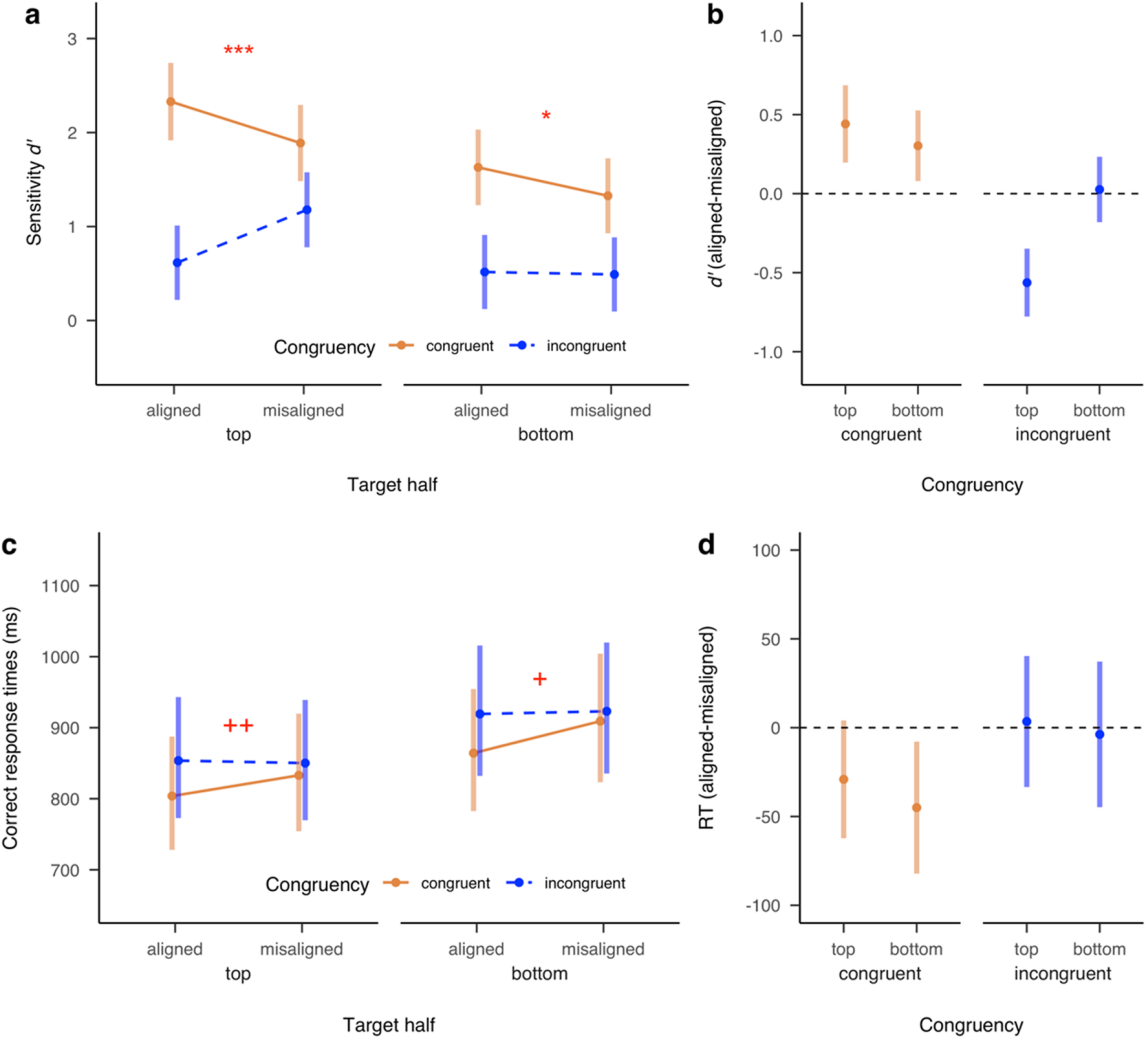
Sensitivity *d*’ (**a, b**) and correct response times (**c, d**) as a function of Target (top vs. bottom), Congruency (congruent vs. incongruent), and Alignment (aligned vs. misaligned) in Experiment 1. Error bars denote the 95% confidence intervals of condition means. Plus and asterisk signs denote the statistical significance of the interaction between Congruency and Alignment. Specifically, + indicates *p* = 0.051; ++ indicates *p* = 0.08; * indicates *p* < .05; *** indicates *p* < .001

Figure 3 illustrates the *d′* and RT results for Experiment 1. When judging either top or bottom targets, the congruency effect was observed for aligned composites, with higher *d′* and shorter RT for congruent than incongruent trials, top: *d′*: β = 1.71, 95% confidence intervals (CI) [1.38, 2.05], *z* = 9.94, *p* < .001, RT: β = −49.9, 95% CI [−76.2, −23.6], *z* = −3.72; *p* < .001; bottom: *d′*: β = 1.11, 95% CI [0.78, 1.44], *z* = 6.58; *p* < .001, RT: β = −55.1, 95% CI [−83.9, −26.3], *z* = −3.75; *p* < .001. Moreover, this congruency effect for aligned composites was larger than for misaligned composites for both top and bottom targets, as indicated by the significant interaction between Congruency and Alignment in *d′*, top: β = 1.00, 95% CI [0.73, 1.27], *z* = 7.29, *p* < .001, bottom: β = 0.28, 95% CI [0.02, 0.53], *z* = 2.13, *p* = .03, although the same interactions were not but approached significant in RT: top: β = −32.6, 95% CI [−69.6, 4.4], *z* = −1.73, *p* = .08, bottom: β = −41.2, 95% CI [−82.6, 0.1], *z* = −1.95; *p* = .0507. These results together indicate that composite effects were found for both top and bottom targets.

Figure 3 also illustrates the *d′* and RT results plotted separately for facilitation versus interference (Panels b and d). Facilitation, as measured by better or faster performance for aligned than misaligned composites in the congruent condition, was observed for both the top and bottom targets, top: significant in *d′*: β = 0.44, 95% CI [0.20, 0.69], *z* = 4.49, *p* < .001, though not in RT: β = −29.1, 95% CI [−62.3, 4.1], *z* = −2.19, *p* = .11; bottom: significant in both *d′*: β = 0.30, 95% CI [0.08, 0.53], *z* = 3.38; *p* = .003, and RT: β = −45.0, 95% CI [−82.2, −7.9], *z* = −3.02, *p* = .01. By contrast, interference, as measured by worse or slower performance for aligned than misaligned in the incongruent condition, was only observed for top targets, significant in *d′*: β = −0.56, 95% CI [−0.78, −0.35], *z* = −6.54, *p* < .001, though not in RT: β = 3.5, 95% CI [−33.4, 40.4], *z* = 0.24, *p* > .99, but not for bottom targets: *d′*: β = 0.03, 95% CI [−0.18, 0.23], *z* = 0.32; *p* > .99, or RT: β = −3.8, 95% CI [−44.8, −37.2], *z* = −0.23, *p* > .99.

### Discussion

Experiment 1 revealed the expected composite effects in the complete composite task for both top and bottom targets. More importantly, although facilitation was observed for both top and bottom targets, interference was found for top targets only. Thus, facilitation and interference do not always occur simultaneously for different target face halves, suggesting that facilitation and interference likely result from different sources and independently contribute to the composite effects in the complete composite task.

We explored the asymmetry between facilitation and interference further and investigated its specificity to participants’ strategies. Since the optimal fixation for face processing is typically located slightly below the eyes (e.g., Peterson & Eckstein, 2012), participants might have adopted the strategy of paying more attention to the top halves, even though the Target condition was evenly distributed for top and bottom halves in Experiment 1. It remains unclear whether the observed asymmetry between facilitation and interference in Experiment 1 was specific to this natural viewing strategy or similar asymmetry could be observed regardless of adopted strategies. In Experiment 2, we manipulated the probability of cueing the top targets, in response to which participants were assumed to adopt different strategies, to examine whether that affected facilitation and interference differently.

## Experiment 2

### Methods

#### Participants

With the same statistical power analysis conducted in Experiment 1, another group (32 participants in total, 26 females and 6 males, mean age = 21.13 years) of 29 Chinese participants from the University of Hong Kong and 3 East Asians from the New York University Abu Dhabi with the same criteria from Experiment 1 were recruited in Experiment 2 in exchange of 120 HKD or 100 AED.

#### Stimuli, apparatus, and procedure

All stimuli, apparatus, and procedure were identical to those in Experiment 1, except that instead of distributing the Target condition evenly for the top versus bottom halves in one session, each participant completed two sessions where the probability of the top halves being the targets was either 25% or 75% (the bottom halves were the targets for the remaining trials). The order of the two sessions was counterbalanced across participants, and the interval between the two sessions was 30 minutes to one week. In addition, participants from the New York University Abu Dhabi sat about 57cm away from a 24-inch monitor with the refresh rate of 60 Hz. The aligned and misaligned composite faces subtended 5.60° × 7.25° and 8.40° × 7.25° of visual angle, respectively, which were similar to the setting used at the University of Hong Kong.

This experiment involved five within-subject factors: Probability of cueing top (25% vs. 75%), Target (top vs. bottom), Congruency (congruent vs. incongruent), Alignment (aligned vs. misaligned), and Correct Response (same vs. different). There was a total of 640 trials in each session, with 40 and 120 trials in each condition for the 25% and 75% cued halves, respectively. Each session lasted around 50 min. Prior to each session, participants completed 32 practice trials with the same cueing probability for the specific session.

#### Data analysis

Data analysis was identical to Experiment 1, except that an additional factor, Probability of cueing top (Probability: 25% vs. 75%), and its interactions with other factors were also included in GLMM as fixed and random effects. To examine the presence of facilitation or interference, Sidak methods of eight estimates were applied. No data were excluded.

### Results

**Fig. 4:**
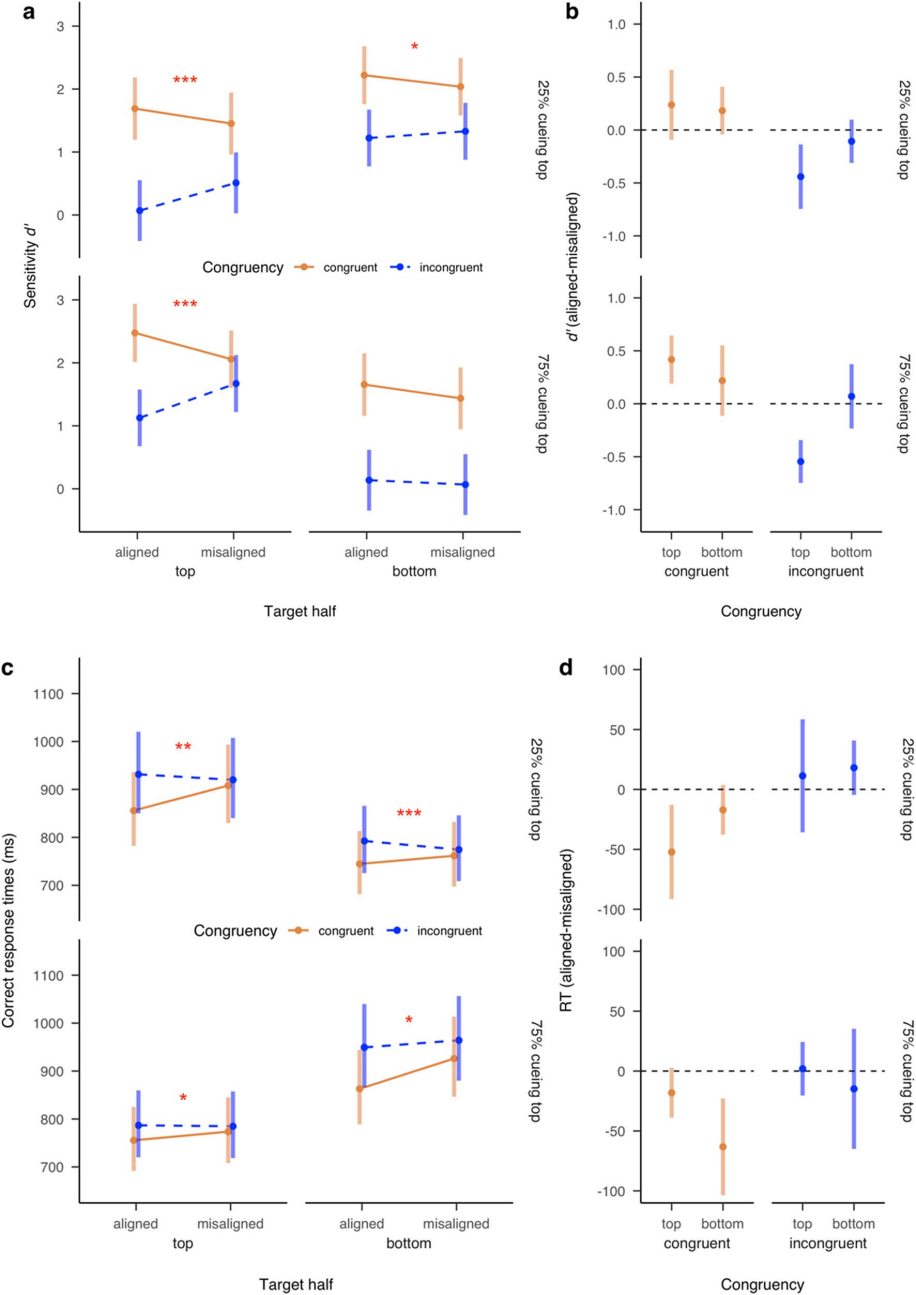
Sensitivity *d′* (**a, b**) and correct response times (**c, d**) as a function of Probability of cueing top (25% vs. 75%), Target (top vs. bottom), Congruency (congruent vs. incongruent), and Alignment (aligned vs. misaligned) in Experiment 2. Error bars denote the 95% confidence intervals of condition means. Asterisks denote the statistical significance of the interaction between Congruency and Alignment. Specifically, * indicates *p* < .05; ** indicates *p* < .01; *** indicates *p* < .001

Figure 4 illustrates the *d′* and RT results of this experiment. When judging either top or bottom targets in either cuing condition, the congruency effect was observed for aligned composites. For top targets, the congruency effect was observed for 75% top-cueing in *d′*: β = 1.35, 95% CI [0.99, 1.71], *z* = 7.42, *p* < .001, and RT: β = −31.3, 95% CI [−46.4, −16.2], *z* = −4.06; *p* < .001, and for 25% top-cueing in *d′*: β = 1.62, 95% CI [1.23, 2.01], *z* = 8.06, *p* < .001 and RT: β = −75.5, 95% CI [−107.2, −43.8], *z* = −4.67, *p* < .001. For bottom targets, the congruency effect was also observed for 75% top-cueing in *d′*: β = 1.52, 95% CI [1.12, 1.91], *z* = 7.53, *p* < .001 and RT: β = −86.4, 95% CI [−118.8, −54.1], *z* = −5.24, *p* < .001, and for 25% top-cueing in *d′*: β = 1.00, 95% CI [0.64, 1.35], *z* = 5.48, *p* < .001, and RT: β = −47.9, 95% CI [−63.4, −32.3], *z* = −6.03, *p* < .001. Moreover, the congruency effect for aligned composites was larger than for misaligned composites as indicated by the significant interaction between Congruency and Alignment observed for all the target and cueing conditions in either *d′* or RT, or both. For

top targets, the interaction was significant for both 75% top-cueing in *d′*: β = 0.96, 95% CI [0.73, 1.19], *z* = 8.15, *p* < .001, and RT: β = −20.0, 95% CI [−39.7, −0.33], *z* = −1.99, *p* = .046, and for 25% top-cueing in *d′*: β = 0.68, 95% CI [0.35, 1.01], *z* = 4.00, *p* < .001, and RT: β = −63.6, 95% CI [−106.4, −20.8], *z* = −2.91, *p* = .004. For bottom targets, the interaction was significant both for 75% top-cueing in RT: β = −48.4, 95% CI [−92.9, −3.83], *z* = −2.13, *p* = .033, though not in *d′*: β = 0.15, 95% CI [−0.19, 0.48], *z* = 0.87, *p* = .38, and for 25% top-cueing in *d′*: β = 0.29, 95% CI [0.06, 0.52], *z* = 2.46, *p* = .01, and RT: β = −35.2, 95% CI [−55.1, −15.2], *z* = −3.45, *p* < .001. These results indicate that when both *d′* and RT were considered, the composite face effects were generally observed in all conditions.

Figure 4 also illustrates the *d′* and RT results plotted separately for facilitation versus interference (Panels b and d). Facilitation, as revealed by better or faster performance for aligned than misaligned composites in the congruent condition, was observed for top targets in 75% top-cueing condition in *d′*: β = 0.42, 95% CI [0.19, 0.64], *z* = 5.02, *p* < .001, though not in RT: β = −18.1, 95% CI [−38.9, 2.7], *z* = −2.38; *p* = .13, and for top targets in 25% top-cueing condition in RT: β = −52.2, 95% CI [−91.6, −12.9], *z* = −3.62; *p* = .002, though not in *d′*: β = 0.24, 95% CI [−0.09, 0.57], *z* = 1.96, *p* = .34. Facilitation was also observed for bottom targets in 75% top-cueing in RT: β = −63.2, 95% CI [−103.5, −22.9], *z* = −4.28, *p* < .001, though not in *d′*: β = 0.22, 95% CI [−0.11, 0.55], *z* = 1.79, *p* = .46. However, no facilitation was observed for bottom targets in 25% top-cueing condition, either in *d′*: β = 0.18, 95% CI [−0.04, 0.41], *z* = 2.22, *p* = .19, or RT: β = −17.1, 95% CI [−37.7, 3.6], *z* = −2.25; *p* = .18. More importantly, facilitation was greater for facial halves when they were less cued; RT: top targets in 25% vs. 75% top-cueing, β = −34.13, 95% CI [−65.06, −3.19], *z* = −2.16; *p* = .03; bottom targets in 25% vs. 75% top-cueing, β = 46.18, 95% CI [14.67, 77.70], *z* = 2.87; *p* = .004; though not in *d′*, *p* > .20.

By contrast, interference, as revealed by worse or slower performance for aligned than misaligned composites in the incongruent condition, was observed only for top targets, for both 75% top-cueing in *d′:* β = −0.55, 95% CI [−0.75, −0.34], *z* = −7.35, *p* < .001, and for 25% top-cueing in *d′*: β = −0.44, 95% CI [−0.74, −.14], *z* = −3.95, *p* < .001, though no significant interference for top targets was observed in RT (*p*s > .99). For bottom targets, no significant interference was observed either in *d′* (*p*s > .73) or RT (*p* > .98 for 75% top-cueing;* p* > .21 for 25% top-cueing). Different from the results of facilitation, no significant differences of interference were observed for both targets between 25% and 75% top-cueing conditions (*p* > .09).

These results suggest that for top targets, significant facilitation and interference were observed regardless of target cueing probability. However, for bottom targets, facilitation was only occasionally observed in one of the target cueing conditions (only in 75% top-cueing), and no significant interference was observed.

### Discussion

Experiment 2 revealed the expected composite effects for both top and bottom targets regardless of the target cueing probability. Importantly, whereas facilitation was found for all conditions except for bottom targets when they were more cued, interference was consistently observed for top targets only, irrespective of target cueing probability. This finding suggests that facilitation, but not interference, is likely affected by participants’ strategies. Also, facilitation and interference do not always occur simultaneously for different target face halves with distinct cueing probabilities. These results continue to indicate facilitation and interference contribute differently to the composite effects measured by the complete composite task.

## General Discussion

Our study examined facilitation and interference in one paradigm (i.e., the complete composite task) to exclude the impacts of potential confounds of task formats and response bias. As noted above, facilitation and interference refer to the influence of irrelevant facial parts on the target parts. Results showed that, first, consistent with previous studies (e.g., Z. Wang et al., 2019), composite face effects were observed for both facial halves and across different probabilities of target locations. Second, facilitation and interference were found in congruent and incongruent trials of the complete composite task respectively, but they varied from each other and were asymmetric.

The standard composite task consists of a subset of trials in the complete composite task, and several studies using the composite task have employed the complete design, but reported results from both measures, as one way to address concerns about task designs (Cheung et al., 2008; Hayward et al., 2016; Jin et al., 2022; Richler & Gauthier, 2014). Notwithstanding other differences, such as the use of accuracy of same trials (standard design) or *d′* (complete design) as the dependent measure, here we show that interference clearly occurs in the complete composite task, as well as facilitation; however, we also show that facilitation and interference do not always co-occur.

In this study, facilitation appears over a broad range of conditions, whereas interference is more likely to occur for top-cued composites only. This asymmetry seems to result from the discrepancy in integrating facilitating and interfering information for identity judgments. Incorporating facilitating information appears flexible in terms of the target position, cueing probability, and measures: both top and bottom facial halves could facilitate the identification of the other parts in most conditions, and facilitation was manifested by more accurate or faster responses. By contrast, integrating incongruent information appears stable: Interference was only observed for identification of the top halves and was typically reflected by worse behavioral sensitivity rather than slower responses. Note that the absence of interference for target bottom halves was unlikely due to a floor effect because the overall performance (*d′*) for the bottom conditions was significantly higher than chance and no interference was observed in any bottom conditions across the experiments.

The overall accuracies in both experiments were around 70%, and this overall accuracy of 70% suggests that sensitivity d’ was an appropriate dependent variable in this study in estimating the effects of the composite face task. By only considering the results from sensitivity *d′*, interference was still consistently observed for top-cued composites only regardless of cueing probabilities. By contrast, facilitation could be observed for the top- or bottom-cued composites, though in distinct cueing probability conditions. It is important to note that since the accuracies varied among conditions, the numbers of correct trials in the various conditions were not identical for response time analysis on the correct trials. Although there is a potential concern that the unbalanced number of correct trials might threaten the validity of the RT analysis of correct trials, we found that consistent conclusions could be drawn based on both sensitivity d’ and correct RT results. Specifically, regardless of whether evidence is used from just sensitivity *d′*, or is supplemented with the RT analysis, the asymmetry between facilitation and interference was observed.

We speculated the asymmetry between facilitation and interference might result from their differences in perceptual fields (see Rossion, 2009): the perceptual fields for facilitation might be similarly large for both top and bottom targets, whereas those for interference might be larger for top relative to bottom targets (e.g., Z. Wang et al., 2023). It is worth noting that the asymmetric facilitation and interference do not necessarily stem from distinctive neural mechanisms. For instance, they may reflect two dissimilar behavioral effects stemming from common spatial integration in face-selective cortical regions (Poltoratski et al., 2021). The specific divergent mechanisms leading to the dissimilar behavioral effects should be explored further. For instance, gaze-contingent paradigms could be employed with computational modeling to explore the differences in perceptual fields for facilitation and interference, and their linkage to the population receptive fields of face-selective cortical regions.

Since it seems that facilitation and interference can also be observed in the part–whole and standard composite tasks, how do such effects correspond to the facilitation and interference observed in the complete composite task? In the standard composite task, the composite effect (interference) is estimated by the performance difference between aligned and misaligned conditions where the top halves are identical and the bottom halves are different. This condition corresponds to the same-incongruent condition in the complete design (Fig. 1C). By contrast, the effects measured by the different-incongruent trials are less clear. In this condition, the same bottom facial halves may make the different top halves look more similar or have no impact. In any case, it is unlikely that the same bottom half renders the different top halves more different. Thus, it seems clear that the incongruent trials in the complete composite task and standard composite task, although not identical, measure the same aspect of holistic face processing (i.e., interference).

Typically, the part–whole paradigm employs a two-alternative forced-choice task: after learning a study face, participants are shown the original face and a foil face, of which a key facial feature is replaced. Critically, the study-original pair and study-foil pair of whole faces correspond to the same-congruent and different-incongruent trials in the complete composite task (Fig. 1C), which reflect facilitation and interference, respectively. However, given an advantage for whole faces over facial features is consistently observed, the part–whole effect may more likely reflect facilitation than interference. Some potential insights can also be obtained from Li and colleagues (Li et al., 2017, 2019), who provided evidence that distinctive neural mechanisms underlay the standard composite and the part–whole tasks. Considering that the standard composite face task may more likely measure interference, it may be that the part–whole task largely reflects facilitation. The specific contribution of facilitation and interference to the part–whole effect can be further explored.

In this paper, we observed that facilitation and interference are asymmetric in holistic face processing. As discussed earlier, the standard composite effect likely reflects interference whereas the part–whole effect appears based more heavily on facilitation. Considering that facilitation and interference are asymmetric as observed in this study, the lack of associations between these paradigms reported in previous literature (Boutet et al., 2021; Rezlescu et al., 2017; Wang et al., 2012) are unsurprising. One limitation here was that we tested facilitation and interference in only one paradigm to minimize the influence of potential confounds, but did not test them in other paradigms. Therefore, we must be cautious in generalizing the facilitation and interference in the complete composite task to the effects observed with the standard composite task and the part–whole task directly. Future studies can discern facilitation and interference tested in the part–whole and other paradigms, and may inspect relationships among the different components via individual differences. The understanding of facilitation and interference may also benefit from examining the contributions of other cognitive or decision-making components underlying face processing. These efforts have the potential to disentangle mixed findings from different popular paradigms measuring holistic processing in one unified framework. All in all, we characterize facilitation and interference as reflecting two asymmetric effects of holistic face processing; a clear account of these different effects will be necessary to explain the nature of holistic face processing.

## Data Availability

Data are available online (https://osf.io/yhm3s/). Materials are not available due to copyrights.

## Footnotes

1 Note that two common versions of this task, which we will call the “standard” design (e.g., Rossion, 2013) and the “complete” design (e.g., Richler & Gauthier, 2014), are differentiated in Fig. 1.

2 DeGutis et al. (2013) employed both subtraction and regression to calculate holistic processing effects. For subtraction, the holistic processing effect was calculated as the performance in experimental condition subtracting that in the baseline condition. For regression, the performance in the experimental condition and that in the control condition were used as the dependent and independent variables in the linear model, respectively, where the holistic processing effect is indexed by the residuals.

3 Although the analysis of correct RT is the standard practice, the analysis of RT in all trials showed highly similar results.

## Code availability

Data analysis code is available online (https://osf.io/4ecjz/).
